# Myxospore density of *Kudoa inornata* varies significantly within symmetrical white muscle tissue replicates of its fish host, the spotted seatrout, *Cynoscion nebulosus*

**DOI:** 10.1007/s00436-024-08333-8

**Published:** 2024-09-05

**Authors:** Augustus M. Snyder, Eric J. McElroy, Isaure de Buron, Fabio Casu, Jody M. Beers

**Affiliations:** 1 Department of Biology, College of Charleston, Charleston, SC 29412 USA; 2 National Institute of Standards and Technology, 331 Ft Johnson Rd, Charleston, SC 29412 USA; 3 South Carolina Department of Natural Resources, Marine Resources Research Institute, Charleston, SC 29412 USA; 4 Institute of Arctic Biology, University of Alaska Fairbanks, Fairbanks, AK 99775 USA

**Keywords:** *Kudoa*, Seatrout, Myxozoa, Sampling variation, Parasite density, Sciaenidae

## Abstract

The spotted seatrout, *Cynoscion nebulosus*, is a popular game fish in the southeastern USA. It is estimated that nearly 90% of the adult population in South Carolina estuaries are infected in their skeletal muscle by the myxosporean, *Kudoa inornata*. However, little is known about this parasite’s biology, including the distribution and densities of myxospores within tissues of infected fish, which we expect affect the physiology of their hosts. In order to correlate densities with physiological parameters in future studies, we quantified the myxospores density in muscle and characterized the variation among individual fish. Naïve juvenile seatrout was experimentally infected via presumed *K. inornata* actinospores exposure to raw seawater. A plug of muscle was extracted from two bilaterally symmetrical regions in the epaxial fillet from fresh and frozen carcasses. Variation in density data was calculated both within and among individuals. Within individuals, density counts were compared between left- and right-side biopsies. There was no significant difference between fresh and frozen plugs, and variation among individuals accounted for the greatest proportion of variation at 68.8%, while variation within individuals was substantial at 25.6%. Simulation and correlation tests confirmed that bilaterally symmetrical replicates varied significantly within individuals. When sampled from areas surrounding the initial biopsies, myxospore density estimates were more similar than between sides. Our findings have important implications for sampling design, particularly for studies investigating physiological parameters at the cellular or molecular level in association with parasite infection.

## Introduction

The use of anatomically symmetrical replicates is a widespread practice among physiologists in various disciplines, but in some cases, it could lead to concerns of pseudo-replication. It is well-known that many physiological parameters (e.g., cell type and density, enzyme activity, body temperature, and pH) may vary with respect to sampling location in tissues (Hamilton et al. 2020)—even within individual organisms—and for this reason, sampling methodology is a critical consideration for study design. This consideration applies to studies that would attempt to describe how parasites affect their hosts at the cellular and molecular levels as all could vary widely depending on various interacting factors, such as host immunological response (Antia et al. 1996), temperature (Burnett 1953; Kirk et al. 2018; O’Connor and Bernhardt 2018), exposure time (Stadler et al. 2023), as well as measurement techniques (Hammami et al. 2013).

Myxosporeans are microscopic cnidarians, whose myxospores infect a variety of vertebrates, of which fish are among the most common (Hallett et al. 2015). Although myxospores have an intimate relationship with their hosts, to our knowledge, little appears to be known regarding their distribution within fish hosts, especially with respect to differences between left and right sides of the fish. Along the Atlantic coast, the spotted seatrout, *Cynoscion nebulosus* (Cuvier, 1830), is an attractive model organism for physiological studies of host-parasite relationship. This fish is an abundant and highly sought recreational species, and adults were reported to be commonly infected by the myxosporean *Kudoa inornata* Dyková et al. 2009 (Arnott et al. 2017). Although not the case for all species of skeletal muscle dwelling species of *Kudoa* (see Kabata and Whitaker 1981; Kudo et al. 1987; Dawson-Coates et al. 2003), cranial and epaxial regions are generally considered to be the least-variable areas for sampling muscle to determine the infection status of a given fish (e.g., Oliva et al. 1992; Henning et al. 2013). While distribution of *Kudoa* in fish muscle has been reported to be homogeneous in the anterior and central body regions of the host fish somatic muscles for two species (Oliva et al. 1992; Ware et al. 2014), this notion cannot be generalized. Our preliminary observations in this regard suggested that, while plasmodia may be homogenous in the epaxial fillet of seatrout, myxospore density appears to vary significantly within fish but without a discernible or consistent trend. This is in line with other studies that showed high variability of parasite density among individual fish infected by *Kudoa* spp., regardless of the methods employed to quantify myxospore density or the species studied. For example, Kawai et al. (2012) and Yokoyama et al. (2015) reported highly variable myxospore densities for *K. septempunctata* Matsukane, Sato, Tanaka, Kamata & Sugita-Konishi, 2010 in Olive flounder *Paralichthys olivaceus* (Temminck and Schlegel, 1846) individuals using a hemocytometer, with values spanning three orders of magnitude and ranging from ~ 10^4^ to 10^7^ spores g^−1^-muscle tissue. Likewise, despite employing an enzymatically based myxospore liberation process optimized for a different species, McElroy et al. (2015) report comparable variation in their inter-individual myxospore density estimates in seatrout infected by *K. inornata*, ranging from ~ 10^4^ to 10^8^ spores g^−1^. Similarly, a study that employed molecular quantitative PCR techniques to estimate *Kudoa thyrsites* myxospore density in Atlantic mackerel reported highly variable data that span two or more orders of magnitude (Giulietti et al. 2022). Overall, each method currently employed to quantify density of *Kudoa* appears to show similar degrees of variability in data acquired, suggesting a biological rather than methodological origin and thus far interpreted as such by all authors.

Based on this information, we chose to sample epaxial muscle using myxospore extraction and tabulation methods according to McElroy et al. (2015) in seatrout/*K. inornata* system studies. However, we desired to maximize the number of assays and experiments that could be performed from a limited sample availability. This led us to investigate whether anatomically matched sampling regions in the epaxial fillet, but from opposite sides of the fish, could be used to estimate parasite density. Thus, the goals for this study were threefold: (1) quantify the proportion of variance in myxospore density measurements among and within seatrout individuals; (2) test whether the ranking of individuals’ muscle myxospore densities was consistent between the left and right sides of the fish; and (3) test whether tissue sampling of the area surrounding biopsy sites may be used to attain high-resolution and accurate measurements of local parasite density. We expected myxospore density estimates to differ significantly with respect to sampling location within individuals but for the differences among fish and their rankings (i.e., relative order of myxospore densities) to remain reliable and consistent.

## Materials and methods

### Animal husbandry

Juvenile seatrout (approximately 30 days old, averaging a total length of 36 mm and a weight of 0.33 g) were acquired from the Waddell Mariculture Center (Bluffton, South Carolina [SC]) and transported to the Department of Natural Resources (SCDNR) Marine Resources Research Institute in Charleston, SC, USA. Fish were initially kept indoors in tanks receiving settled harbor seawater (SHS), which is water that has been held in a tower and allowed for particulates to settle for at least 72 h before delivery to tanks. Fish reared on SHS are known to acquire little to no infection by *K. inornata* (de Buron, unpublished data). After a period of ~ 30 days, approximately 350 individuals were moved to outdoor rearing tanks receiving raw Charleston Harbor seawater (RHS) known to carry the infective stage of *K. inornata* (presumed actinospores) (de Buron et al. 2017). Various numbers of fish (either 25 or 75 individuals per tank) were held in four 1.83-m diameter (~ 1000 L) tanks that varied in flow rate, receiving either 16.5 or 61.5 L min^−1^ of water, to obtain a range of myxospore densities within individuals. This is because infection of seatrout by *K. inornata* is believed to be actinospore dose-dependent (de Buron et al. 2017). Additionally, a subset of fish from the same mariculture cohort (and therefore of similar age and body size) were kept on RHS in a 6.83-m tank intended for use in another study. These fish were later distributed into three 1.83-m tanks in roughly equal proportions (approximately 80 fish per tank) at flow rates ~ 30 L min^−1^. Fish used in the present study were obtained from both sources. All fish were given an age-appropriate commercial pellet diet (1 mm, 3 mm, or 5 mm; Zeigler “Finfish G”; 42% protein, 16% fat) and fed to satiation daily to help prevent cannibalism (Manley et al. 2015); fish held outdoors on RHS also fed opportunistically on incoming zooplankton and invertebrates. Water quality parameters including temperature, dissolved oxygen, pH, and salinity were monitored and recorded daily using a YSI Professional Plus (Yellow Springs, Ohio, USA). Due to logistic constraints on indoor tank availability and sampling times, the length of fish exposure to RHS and therefore to actinospores of *K. inornata* was not tightly controlled. Fish from the first group of individuals receiving various flow rates were sampled in July, while fish from the other group of individuals receiving constant flow rates were sampled from October to December, 2020. Fish size and age ranged from 19 to 33 cm and 1 to 1.5 years, respectively.

### Muscle sample collection

**Fig. 1:**
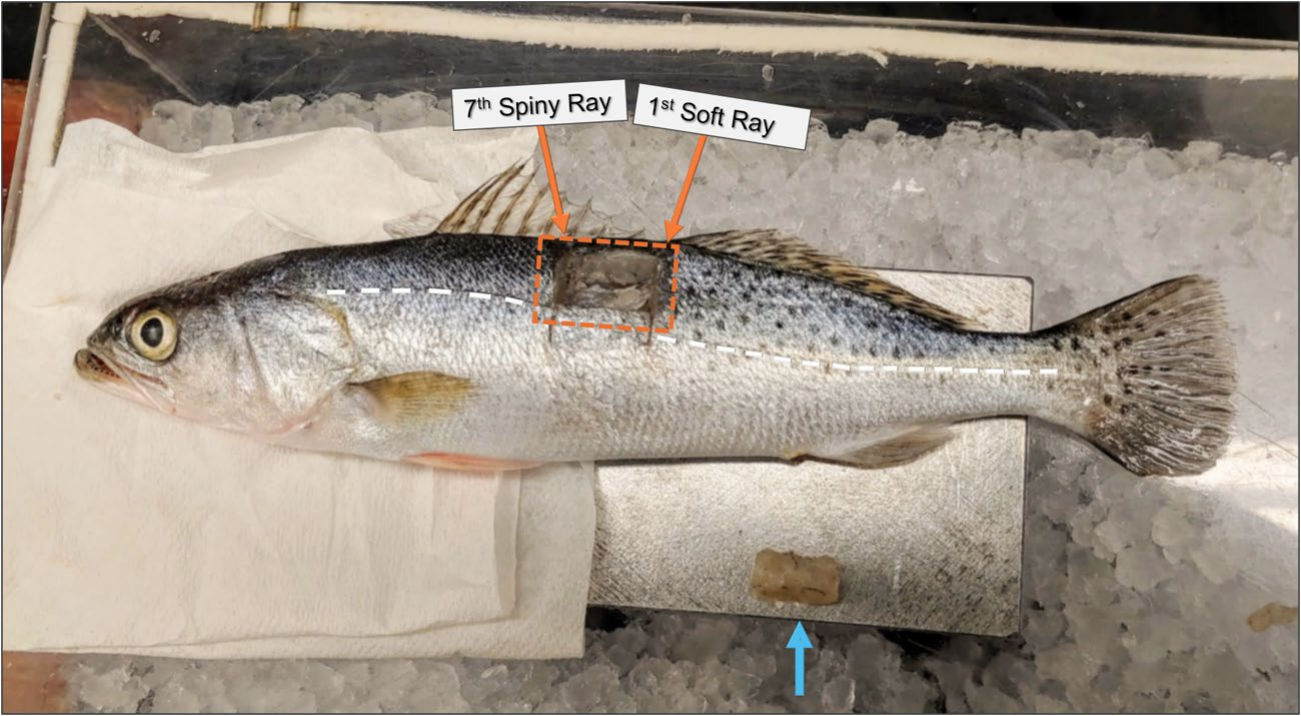
Representative depiction of epaxial white-muscle biopsy (blue arrow) from the left side of a spotted seatrout. Sample location (i.e., 7seventh spiny ray to fir1st soft ray, down to lateral line) is indicated by the scalpel cuts and orange arrows. The lateral line is indicated by white dashed line. The region of tissue sampled from the fish carcasses for supplemental myxospore density counts is indicated by the dashed orange lines. A roughly equal portion of tissue was removed from an identical location on the opposite side of the fish

All fish used in this study were anesthetized in a container with 30-L seawater, 3.4 g MS-222 (anesthetic), and 6.8 g NaHCO_3_ for buffering pH. Fish were then euthanized via cervical transection (IACUC # 2020–004). White muscle tissue samples were excised from each animal and immediately snap-frozen in liquid nitrogen prior to storage at − 80 °C. Glycolytic (white) skeletal muscle was chosen because of tissue abundance, and notably because it has been shown to be one of the least-variable areas to sample *K. inornata* infection in any given fish (Ware et al. 2014). Whole fish carcasses were stored at − 20 °C following tissue extraction and later transferred to − 80 °C for additional spore counts at a later date. The portion of the epaxial muscle that was excised was approximately between the seventh spiny ray and the first soft ray of the dorsal fins from both the left and right flanks, slightly above the lateral line (Fig. 1). Portions of the left-side tissue were also taken from those samples for homogenization and various down-stream physiological work. Due to the nature of the homogenization procedure employed, myxospore density estimates of the left-side tissues were not obtainable for those individuals. However, the left-side biopsies of some fish were usable for myxospore counts, thus affording the analyses presented here.

To supplement existing count data, muscle tissue from the stored fish carcasses was also extracted from areas within a ~ 0.5-cm perimeter surrounding the initial biopsy sites (Fig. 1). These samples were taken while the fish was still frozen (i.e., carcass thawed from − 80 to − 20 °C), then chopped and sub-sampled on a cold aluminum dissecting stage chilled on ice and processed identically to the other samples (see “Muscle digest and hemocytometer counts” section below). Due to logistical constraints of samples consigned to other studies, not all four counts (i.e., left biopsy count, right biopsy count, and surrounding area for both sites) were carried out for each fish. However, this work did afford multiple estimates of myxospore density for some individuals—sourced from both the left and right sides—and those samples were selected for downstream analyses (*n* = 42). In total, 158 myxospore counts and density estimates were performed on 73 fish.

### Muscle digest and hemocytometer counts

Myxospore density counts were performed according to McElroy et al. (2015). In brief, muscle tissue was finely chopped and shredded using two glass plates, a razor, and a scalpel. Approximately 70 ± 3 mg of tissue was collected from each sample in a 2-ml microcentrifuge tube with careful effort taken to subsample well-mixed tissue and digested in 0.5 mL of a 0.5% trypsin solution in phosphate-buffered saline (PBS) for 90 min at 37 °C, or until completely digested.

Following digestion, tubes were inverted several times to assure myxospores were well mixed, and a 10 μL aliquot was pipetted onto a Bright-Line hemocytometer (Hausser Scientific). Myxospore counts were performed on either an Olympus CX31 compound microscope at 200 × total magnification, or a Fisher Scientific Micromaster microscope at 400 × total magnification, depending on equipment availability. The number of myxospores was counted across four grids (each grid 1 mm in area, subdivided into 16 squares) for each sample, and myxospore density (i.e., the number of myxospores per gram of muscle tissue) was determined after taking dilution factor, grid area, and subsample tissue mass into account following Snyder et al. (2022). This procedure was performed always by the same person and repeated twice for each sample. Replicates were then averaged and standard deviation was calculated. To evaluate measurement error due to the hemocytometer itself, as well as the digestion and loading procedure, we determined the intra-class correlation coefficient (ICC) between duplicate measurements in R, as well as the average coefficient of variation (CV) for duplicate measurements for both the initial biopsies (i.e., “Plugs”) and surrounding area (i.e., “SurrArea”) counts. Intraclass correlation coefficient values range from 0 to 1, and a value closer to 1 corresponds to better replicability of sample measurements.

### Statistics

**Fig. 2:**
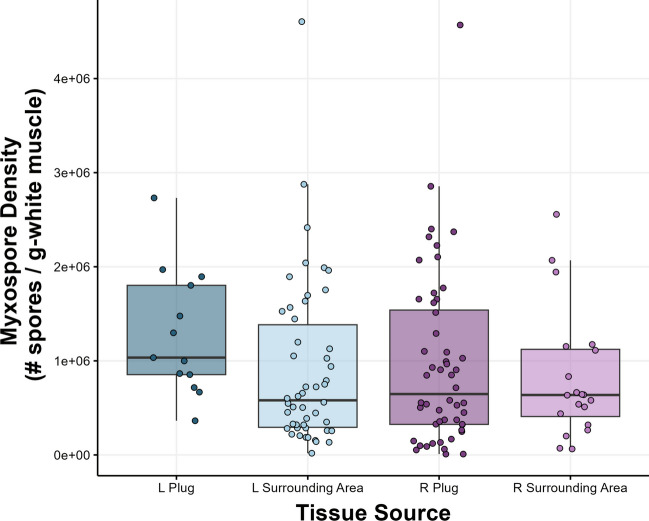
Variation in myxospore density of *Kudoa inornata* between biopsy methods performed on skeletal muscle of spotted seatrout. “L plug” and “R plug” estimates correspond to counts performed on fresh muscle plug tissue excised immediately after euthanasia (L, left; R, right). “L Surrounding Area” and “R Surrounding Area” estimates correspond to counts performed on surrounding tissue taken from the frozen fish carcasses at a later date. Darker colors indicate plug estimates, while lighter colors indicate surrounding area estimates. Boxes represent interquartile ranges (IQRs) of myxospore density estimates for each location sampled; whiskers extend to 1.5*IQRs, while the bold line indicates median estimates. Points represent individual sample myxospore densities

All statistical analyses were conducted in R Studio 2022.02.1 + 461 (R Studio Team, 2022; http://www.rstudio.com/) and R 4.1.3 (R Core Team 2022; https://www.R-project.org/) and used the following packages: ggplot2 (Wickham 2016), patchwork (Pedersen 2022; https://CRAN.R-project.org/package=patchwork), nlme (Pinheiro et al. 2021), scales (Wickham and Seidel 2020), ragg (Pedersen and Shemanarev 2022), performance (Lüdecke et al. 2021), dplyr (Wickham et al. 2023), and psych (Revelle 2024). Each fish was treated as an individual, and statistical significance was inferred when *p* < 0.05. Distributions of myxospore densities from left and right sides of the fish were compared by plotting the counts for each tissue (Fig. 2). A density curve based on count frequency was overlaid for each side to show the continuous nature of these data and to facilitate comparing distributions. To validate the use of tissue surrounding the biopsy sites for estimating myxospore density, data were fit to a model I ANOVA and tested for significant differences in variation. To test the hypothesis that density estimates differed significantly both among and within fish, myxospore density data (*n* = 40 [2 outliers removed]) were log-transformed and fit to a model II nested ANOVA, with tissue location (i.e., left or right fillet) nested within individuals. In total, each of the 40 individuals used in the nested ANOVA model had tissue taken from two sides (left and right), with two counts performed per side. Both “tissue location” and “sample” were treated as random effects. Residual data were leptokurtic, but otherwise passed qualitative checks for model assumptions. The use of a balanced nested ANOVA model enabled post-hoc quantification of the proportion of variance in infection densities, both among and within individuals. This was achieved via algebraic substitution and isolation of the mean square variance components for density estimates of both the “subgroups within group” (i.e., tissue location within individual fish) and “group” (i.e., individual fish) terms (Sokal and Rohlf 1995).

Following the analysis of variance among and within fish groups, the overall significance of the variation of myxospore densities within individuals was investigated using a random resampling test with 1000 iterations via “For-looping” in R. For each of the 40 fish in the nested ANOVA model, one of the two measurements for each side of the fish was selected at random and appended to a new data set, and this was repeated 1000 times for a total of 80,000 rows (i.e., 2000 per individual). After looping, the new larger data set was fit to two mixed-effects linear models using the nlme package in R. Both models included myxospore density estimates as the response variable and tissue location (i.e., left vs. right side) as the treatment variable. One model (ACTUAL) calculated the slopes for each sample with respect to location within fish, and the other model (RANDOM) calculated slopes for each sample randomly. All components of model RANDOM are nested in model ACTUAL, which allowed for an assessment of model fit using AIC score and* p* value from an ANOVA test.

The purpose of the two models described above was to evaluate whether the rank-order of samples could differ significantly depending on whether right or left myxospores density counts are used to estimate density for a given individual. This was further tested using Kendall’s rank-based correlation test of differences between left- and right-side ranks of myxospore densities at two scales, and further visualized via L-rank vs. R-rank correlation plots as well as a parallel coordinates plot.

## Results

### Methodological validation

Myxospore density estimates were overall highly variable and had similar distributions regardless of sampling time (Fig. 2). On average, no significant difference was found between the myxospore density estimates for samples that were taken fresh or from frozen carcasses (one-way ANOVA: *p* = 0.203; *F*_3,131_ = 1.558). The ICC of Plugs in the mixed model “ACTUAL” (with sample nested within location as a random term) was 0.961. We re-ran this test on the raw data to evaluate error in Plugs vs. SurrArea measurements, and ICC values were 0.96 and 0.97, respectively. The average CV for Plugs was 16.3%, while the average CV for SurrArea was 16.6%.

### Distributions of myxospore densities

**Fig. 3:**
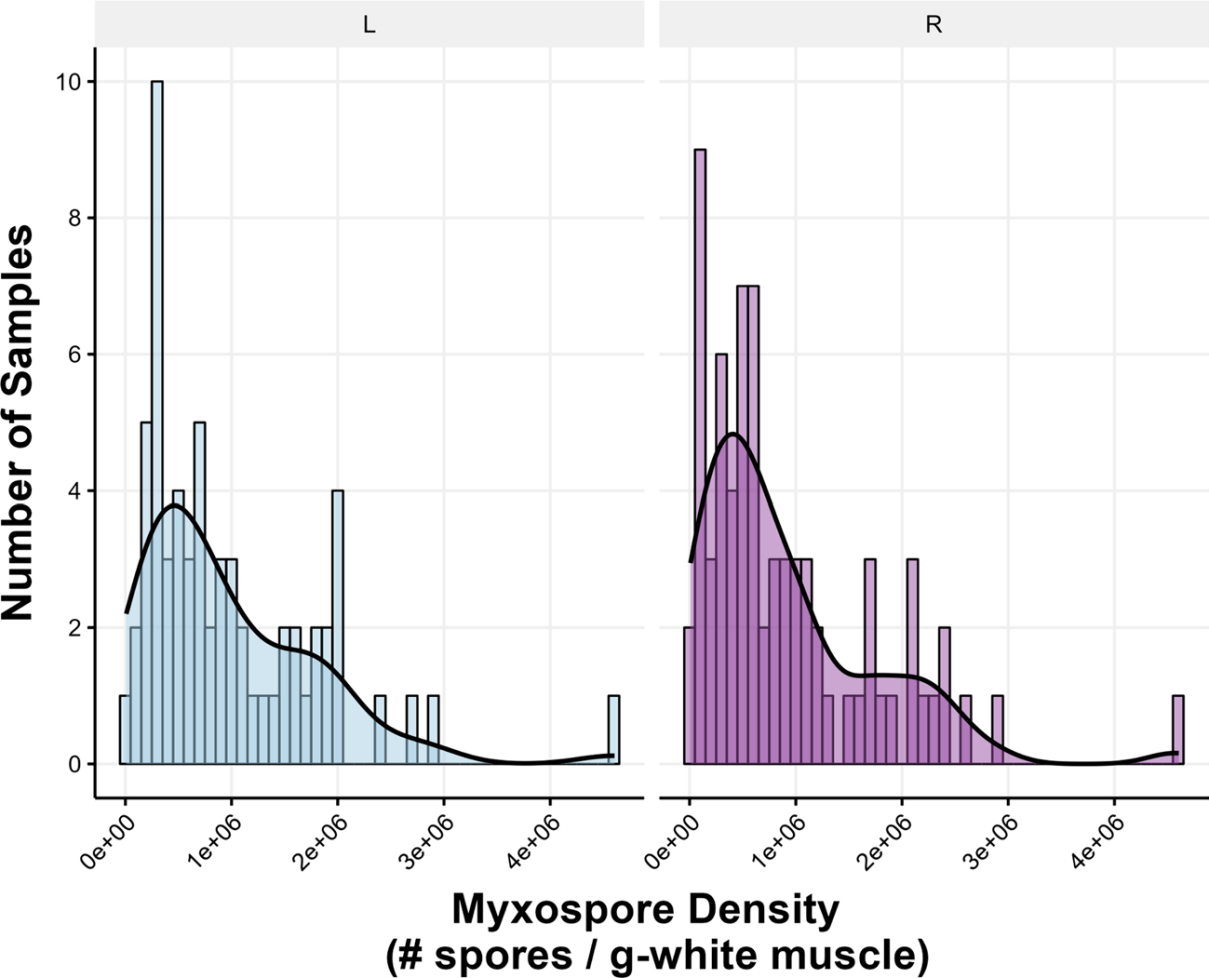
Distribution of myxospore density estimates of *Kudoa inornata* in seatrout white muscle samples (*n* = 73). Colored bars correspond to bins of count data; blue bars indicate samples from the left side of the fish, while purple bars indicate samples from the right side of the fish. Bins are intervals of 100,000 myxospores-per-gram. Uninfected samples are not included. The black line represents the density of count data treated as a continuous distribution from 0 to 5 × 10.^6^ myxospores-per-gram; sum area under the curve = 100% observations (scaled to bin-width)

The estimations of myxospore density in the seatrout examined (*n* = 73 individuals) were highly variable between fish and ranged from 8.93 × 10^3^ spores g^−1^-muscle tissue to 4.61 × 10^6^ spores g^−1^ (Fig. 3). Nine individuals were uninfected and thus removed from further analyses. The distribution of myxospore densities was right-skewed and slightly bi-modal, with large peaks in frequency observed at approximately 5.0 × 10^5^ spores g^−1^, and smaller peaks in frequency between 1.8 and 2.2 × 10^6^ spores g^−1^ (Fig. 3) but appeared nearly identical for both left and right sides. One outlier sample had a very high infection density estimated at 4.6 × 10^6^ spores g^−1^ (for both sides) and was removed from further analyses; all other sample density estimates included in analyses were below 3.0 × 10^6^ spores g^−1^. Another outlier sample removed was estimated to be of extremely low infection density, with one replicate count including a “0” estimate and thus counts could not be trusted for this individual.

### Variation within and among individuals

**Fig. 4:**
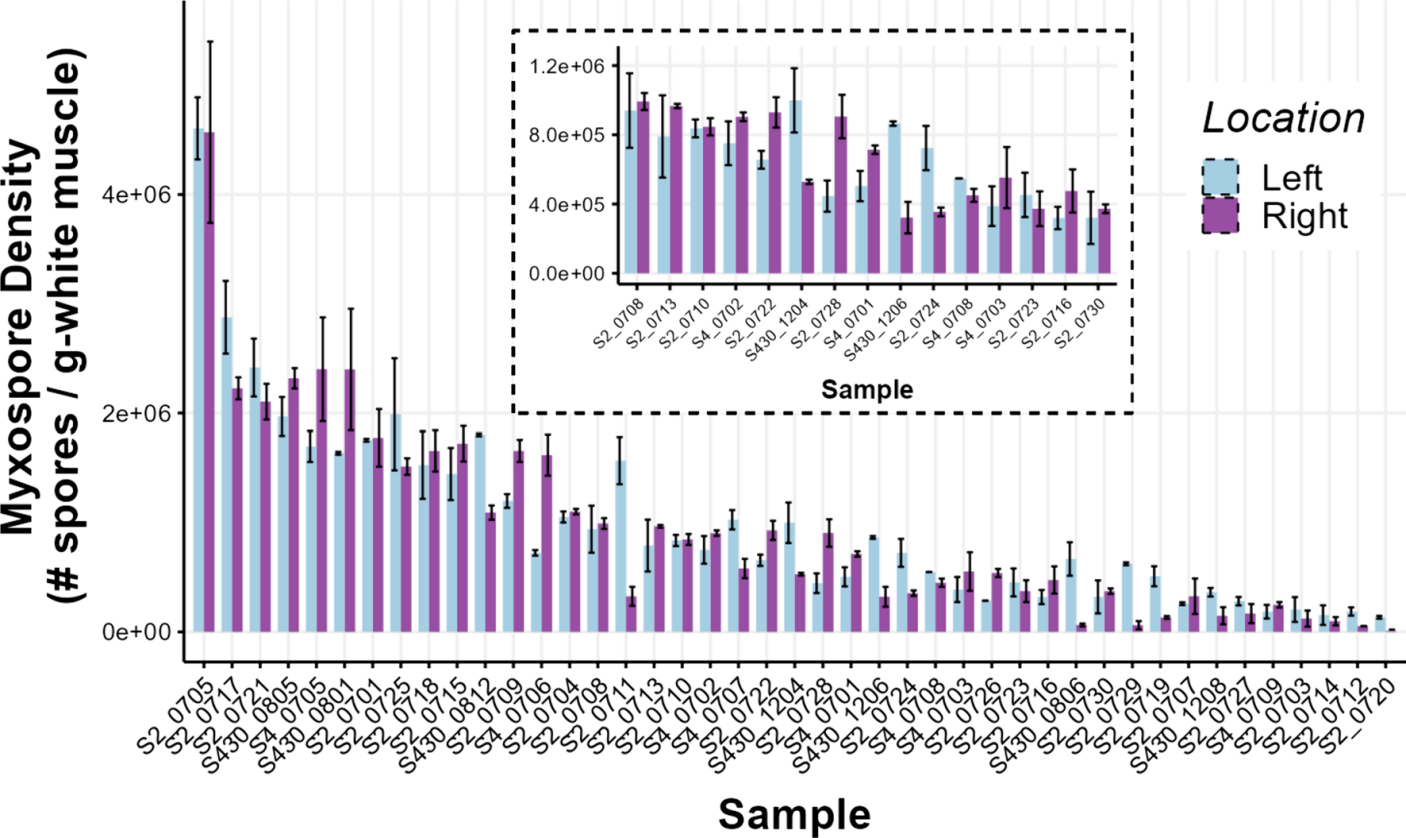
Comparison of myxospore densities of *Kudoa inornata* (left- vs. right-side estimates) within individual seatrout for all samples with available data (*n* = 42). Error bars represent ± 1 standard deviation from the mean (calculated from duplicate counts per sub-sample). Out of the 42 total samples, 40 were used for the nested ANOVA model, and two outlier samples at the highestand lowest infection densities were removed from further analyses. The inlaid plot shows a subset of samples (*n* = 15) for which the left- and right-side density estimates were between 3 × 10^5^ and 1 × 10^6^ spores g.^−1^

**Fig. 5:**
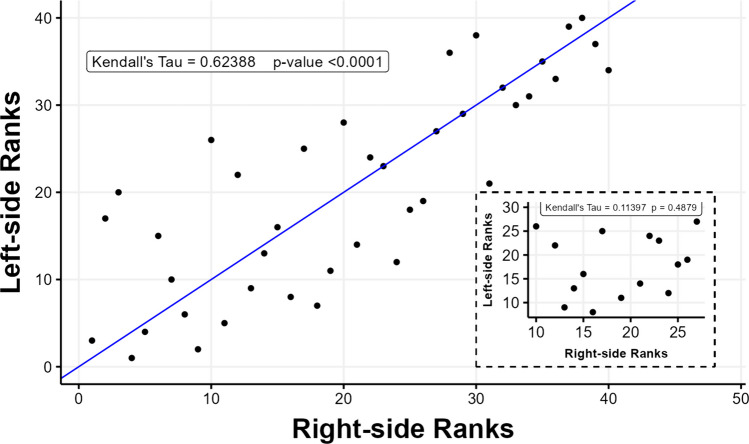
Matched comparison of ranks for left- and right-side myxospore density estimates in seatrout white muscle samples. The blue line represents a line with an intercept of (0,0) and a slope of 1, indicating a hypothetical perfect agreement in sample ranks between sides. The points represent actual samples and their rank-rank coordinates (*x* = right-side rank, *y* = left-side rank). The inlaid plot shows a subset of samples (*n* = 15) for which the differences in the left- and right-side density estimates were between 3 × 10^5^ and 1 × 10^6^ spores g^−1^, matched to the inlaid plot of Fig. 4. Kendall’s correlation test results are indicated accordingly for both sample sets

**Fig. 6:**
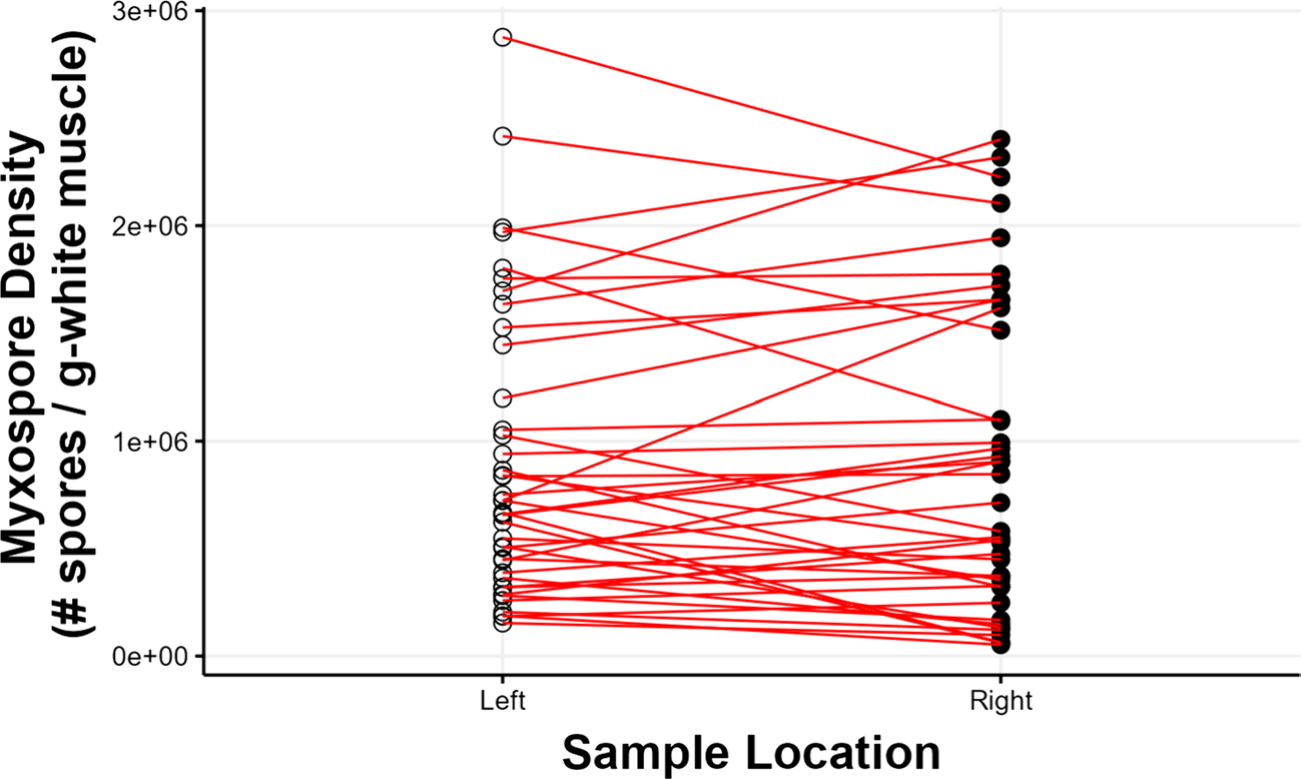
Parallel coordinates plot of left vs. right density estimates. The red lines connect individual samples between their left- and right-side estimates. If the rank-order of samples agreed perfectly for their left- and right-side estimates, the lines would be parallel

**Fig. 7:**
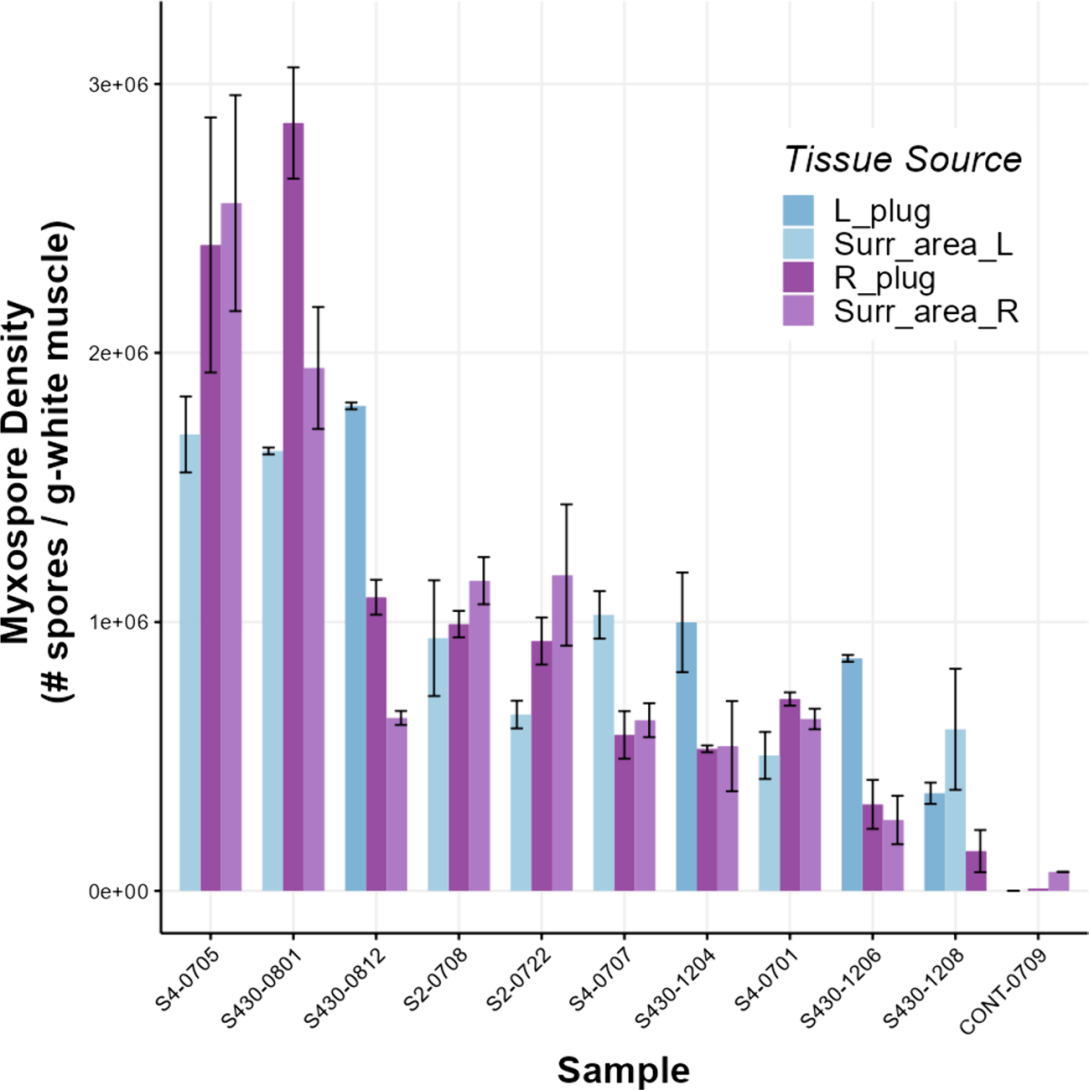
Variation in myxospore density estimates of *Kudoa inornata* within epaxial skeletal muscle of seatrout (*Cynoscion nebulosus*) is greater between sides of the fish than at local scales (within ~ 0.5 cm)

Myxospore densities varied significantly among individuals (Fig. 4; *p* < 0.001; *F*_39,40_ = 5.849), accounting for 68.8% of the variance in myxospore density estimates. Estimates of myxospore density within individuals also varied significantly within fish (*p* < 0.001; *F*_40,80_ = 9.992), accounting for 25.6% of the total variance. Overall residual variation (presumed to be of methodological origin) was low at 5.63%. Additionally, the random resampling test and analysis of variance comparing the mixed effects models showed that ACTUAL model resulted in a significantly better fit than RANDOM model and had a lower AIC score (AIC scores: 2067643 compared with 2185466, respectively) (*p* < 0.0001; ΔAIC score = 117,767, log-likelihood ratio = 117,827). This result indicates that sampling location was important and is consistent with the Kendall’s rank correlation tests of the original data set (Fig. 5). While the range and shape of myxospore density distribution curves appeared nearly identical between sides (Fig. 3), individuals’ myxospore densities differed significantly between left- and right-sides (Figs. 4, 5, and 6), and the rank order was not consistent, especially at the low-end of density between 3.0 × 10^5^ and 1.0 × 10^6^ spores g^−1^ (Fig. 5; *p* = 0.4879; *τ* = 0.11397). At a broader scale, fish ranks were still significantly correlated with each other, and fish whose density estimates were very high or very low tended to have similar left- and right-side estimates (Fig. 5; *p* < 0.0001; *τ* = 0.62387 for all fish). Overall, myxospore density estimates tended to be more similar for regions within 0.5 cm than across sides of a fish (Fig. 7).

## Discussion

In this study, we first characterized the distribution (i.e., the range and frequencies) of density of myxospores of *K. inornata* in glycolytic (white) skeletal muscle for 73 individual seatrout. Nine individuals were uninfected. Of the remaining infected fish, estimates of myxospore density for both left- and right-side tissue were determined for 42 individuals, two of which were outliers. The range of myxospore densities reported here spans two orders of magnitude: 8.93 × 10^3^ spores g^−1^ to 4.61 × 10^6^ spores g^−1^, which is in line with other *Kudoa* spp. studies (Kawai et al. 2012; Yokoyama et al. 2015; Giulietti et al. 2022), including *K. inornata* (see McElroy et al. 2015). Importantly, the study by Giulietti employed RT-PCR for quantification of parasite density, with data in units of parasite DNA mol/mg, so the results may not be directly comparable although the among-sample variability is similar to other studies. Since there was no significant difference in the myxospore densities estimates between fresh and frozen samples, we pooled these samples and proceeded with the nested ANOVA analysis and rank tests. We also observed similar and good replicability of duplicate sample measurements for Plugs vs. SurrArea counts as determined by ICC values ~ 0.96 for both, though average CV was moderate at ~ 16% for both groups. This moderate rate of error indicates that samples with myxospore density estimates that are less than 16% different from each other may not on average be differentiable with our methods and should therefore be taken into account. It is likely that our measurement error could be driven by some samples with relatively low grid cell counts (i.e., ~ 10–100 cells/grid). The standard measurement error for a hemocytometer has been described as 1/$\surd n$ (Berkson et al. 1939) where *n* = no. of cells counted in the hemocytometer. The standard error for a sample with ten cells counted would therefore be 0.316, for example.

As a result of logistical constraints on sampling times, fish that were held in outdoor tanks for longer periods of time likely had greater exposure to actinospores than fish sampled earlier in the year. However, since the peak infectivity of *K. inornata* actinospores occurs during the summer months and is affected by temperature (de Buron et al. 2017), fish sampled from October to December 2020 likely had only slightly greater degree-days of exposure and would have been affected similarly since they were held at similar densities and flow rates. We do not expect that these differences in exposure time would have affected the distribution of *K. inornata* myxospores between left and right sides of the fish within individuals. With respect to fish size, seatrout are reported to reach maturity in the Atlantic between 28 and 35 cm and 1 to 2 years on average but can live to be up to 15 years old and grow to sizes ~ 80 cm or greater (Clardy et al. 2014). Fish size in this study ranged from 19 to 33 cm and fish age ranged from 1 to 1.5 years; thus, we expect any interactive effects with *K. inornata* due to fish age or size in this study to be minimal, though males in the wild appear more likely to acquire infections at a given size or age (Arnott et al. 2017).

With respect to the distribution of sample parasite densities, the overall profiles of the density curves seemed (at first) to be nearly identical for left- and right-side estimates; however, the rank-order of samples was surprisingly not consistent between left- and right-sides. The effect of intra-individual variability on myxospore density appeared more pronounced at the low-to-mid range of infection, such that the rankings of fish with densities between 3.0 × 10^5^ and 1.0 × 10^6^ spores g^−1^ (*n* = 15) were not predictable between the left-side and right-side samples (*p* = 0.4879; *τ* = 0.11397) Notably, this effect appeared random and unbiased (i.e., one side of a fish is not consistently more or less infected). Broadly, however, left- and right-side differences appeared less important for predicting the rankings of fish with very high and very low infection densities when all samples are included (*n* = 40). This suggested that the anatomical sampling side indeed matters for correlative analyses involving fish with moderate infections, but that the usage of fish at extreme densities of myxospores in their muscle would be optimal to correlate potential changes in physiological parameters with infection. In other words, fish with lower myxospore densities have greater intra-individual variability than fish with high myxospore densities (> 1.0 × 10^6^ spores g^−1^), so the ranked order of fish with greater infection loads is likely to be more consistent.

Estimations of myxospore density within seatrout muscle differed significantly, both among- and within-fish. Most (~ 69%) of the variation in the count data was attributable to inter-individual differences, which is likely a reflection of the presumed doses-dependency of infection by *K. inornata* since fish observed were infected at various flow rates and levels of densities in tanks. Fish were also subject to different diets, as some individuals were able to eat opportunistically on small incoming zooplankton and invertebrates in addition to artificial pellets. While the life cycle of *Kudoa* is currently unclear and we cannot rule out possible infection from active feeding on invertebrates (a putative definitive host), we do not believe that this difference in diet affected infection rates or densities between batches of fish because individuals that had access to a live food source had the same range of infection as those fish that ate only artificial pellets. However, there was a discrepancy between the intra-individual myxospores densities differences we observed, and which account for one-quarter of the total variance, and results of Ware et al. (2014) who found that numbers of plasmodia of *K. inornata* were spatially homogenous within fillets from seatrout. Such discrepancy could be explained by the fact that individual plasmodium sizes are highly variable as they progressively grow during myxospore generation, to ultimately fill the entire muscle fibers (Dyková et al. 2009). Therefore, it is possible that, due to biological variability in plasmodia size/maturity and their spatial distribution, the ranked orders of individuals based on their myxospore densities were not perfectly correlative when comparing different regions of the epaxial fillet. Intuitively, tissue samples within ~ 0.5 cm of each other on a given side tended to have more similar myxospore density counts. Such a pattern indicated that densities of myxospores in the fish muscle appeared to be heterogeneous above certain scale thresholds, yet to be determined as they may be defined by a suite of biological factors yet unknown for *K. inornata* (e.g., dose of actinospores, port of entry, cues to select white muscle as site of infection).

Henning et al. (2013) discussed benefits and drawbacks of various methods to recognize and quantify myxospores of *Kudoa*. To our knowledge, there have been no studies to date which have investigated intra-individual variability in host myxospore density for *Kudoa* infections. However, without any ground-truth protocol for determining myxospore density in host tissues, it remains difficult (if not impossible) to estimate and compare count accuracies among the various methods that are commonly used. Significantly however, the proportion of variance due to methodological differences in this study was relatively small (5.6%) even though multiple hemocytometers, different microscopes and magnifications, and slight differences in digestion times among estimates were used. Therefore, the primary source of the large variability in myxospore density data reported here was most likely attributable to biological differences among and within fish/parasites interactions. With respect to myxosporean prevalence, molecular techniques like PCR could be used to detect early or immature parasite stages which are otherwise undetectable via light microscopy (such as pre-sporogonal blood stages or free-living water stages; Hallett and Bartholomew 2006; de Buron et al. 2017). Quantitative PCR may be promising for measuring parasite density more accurately but has yet to be optimized in the case of *K. inornata*. Giulietti et al. (2022) reported densities of *K. thyrsites* DNA (Gilchrist, 1923) in Northeast Atlantic mackerel (*Scomber scombrus* Linnaeus, 1758) using those techniques and showed similar variability in parasite density among fish to other *Kudoa* studies which used microscopy. Hence, molecular methods may not be necessarily better suited to estimating density of mature parasite stages than traditional microscopy, and when one desires to assess the impact of the density of these stages on host physiology, microscopy techniques can work well. However, while early blood stages, immature unsporulated plasmodia, and immature spores may have important impacts on the host in terms of immune response, these stages are difficult to detect with microscopy methods, and thus these techniques are not well suited to assessing the impact of early parasite stages on host physiology. When fish are sampled multiple times and from various anatomical locations as shown here, traditional microscopic techniques may yet be the most reliable to obtain more accurate organismal-level estimates of infection parameters.

Future studies could focus on modeling the spatial distribution (in 2D and/or 3D) of microscopically detectable myxosporean stages in muscle tissue and blood to gain a better understanding of within-fish variability. More importantly however, understanding the mechanisms that underlie the heterogeneity of myxospore density within individuals would allow further use of controlled experiments when studying this host/parasite system. For experiments focusing on interactions at smaller scales, such as in metabolomics studies, we recommend that correlations between various physiological parameters and infection parameters be made using estimates from within ~ 0.5 cm of sampling site or from the same homogenate if protocols allow, to ensure minimal spatial variation in myxospores’ density and maximal resolution of parasite effects. When attempting to relate physiological measurement(s) and parasite burden (e.g., intensity or density according to Bush et al. 1997), reducing spurious methodological variation is paramount. Simply put, resolving host-parasite physiological interactions is difficult and requires careful attention to the biology and tissue-specific responses of both organisms. Given the increasing difficulty in sampling or infecting experimentally statistically appropriate numbers of vertebrate hosts, researchers who want to address the scope of this interplay between parasite and host may decide to maximize their sampling units by taking anatomically similar tissue replicates from a host for use in separate experiments. Provided factors of interest do not vary significantly between these replicates, this experimental design is surely sound and good practice. However, if anatomically symmetrical replicates are not equivalent or functionally homogeneous within an individual, with respect to either parasite burden or some physiological parameters, such replicates may lead to erroneous conclusions.

## Conclusions

In conclusion, we found that myxospore density of *K. inornata* varied significantly within and among individual seatrout. Approximately one-quarter of the total variance was attributable to intra-individual differences, whereas over two-thirds of the variance was attributable to inter-individual differences. Variation attributable to methodological differences was comparatively small (5.6%) in this study. Methods used to quantify Kudoa density vary throughout the literature, but direct counts via microscopy techniques, such as those used here, are most common. These methods, while not sensitive to early blood-parasite stages, immature unsporulated plasmodia, and immature spores, remain valid for estimating density of mature myxospores. We also showed that sampling design could significantly affect results when using myxospore densities for correlative analyses, as predictive power in ranking of samples can change substantially depending on the sampling region within the epaxial fillet, especially at low- to mid-ranges of infection. This is particularly important, given that this was identified by previous authors as the least variable region for sampling seatrout muscle for density studies of *K. inornata*. As a result, we suggest that, for work at cellular and molecular scales, tissue samples used to estimate myxospore density be taken from epaxial regions close (within ~ 0.5 cm or less) of samples used to estimate other parameters. Results from this investigation could be used to help to standardize current methodology for estimating myxospore densities in fish tissues and may yield insights into best practices for researchers designing future studies involving *Kudoa* spp. and possibly histozoic myxosporeans in general.

### Disclaimer

Any mention of commercial products is to specify adequately the analytical procedures used. It does not imply recommendation or endorsement by NIST, or that the products mentioned are necessarily the best available for the intended purpose.

## Data Availability

The raw datasets generated by the current study and the analysis scripts used are available at https://github.com/Augustus-M-Snyder/Variation-in-myxospore-density.

## References

[CR1] Antia R, Nowak MA, Anderson RM (1996). Antigenic variation and the within-host dynamics of parasites. Proc Natl Acad Sci USA 93:985-989 10.1073/pnas.93.3.985 PMC40016 8577773

[CR2] Arnott SA, Dyková I, Roumillat WA, de Buron I (2017) Pathogenic endoparasites of the spotted seatrout, *Cynoscion**nebulosus*: patterns of infection in estuaries of South Carolina, USA. Parasitol Res 116:1729–1743. 10.1007/s00436-017-5449-3 28466246 10.1007/s00436-017-5449-3

[CR3] Berkson J, Magath TB, Hurn M (1939) The error of estimate of the blood cell count as made with the hemocytometer. Am J Physiol - Legacy Content 128:309–323

[CR4] Burnett T (1953) Effects of temperature and parasite density on the rate of increase of an insect parasite. Ecology 34:322–328. 10.2307/1930899

[CR5] Bush, AO, Lafferty KD, Lotz JM, Shostak AW (1997) Parasitology meets ecology on its own terms: Margolis et al. revisited. J Parasitol 83(4):575–583. 10.2307/3284227 9267395

[CR6] Clardy SD, Brown-Peterson NJ, Peterson MS, Leaf RT (2014) Age, growth, and reproduction of Southern Kingfish (*Menticirrhus**americanus*): a multivariate comparison with life history patterns in other sciaenids. Fish Bull 112:178–197. 10.7755/FB.112.2-3.6

[CR7] Dawson-Coates JA, Chase JC, Funk V, Booy MH, Haines LR, Falkenberg CL, Whitaker DJ, Olafson RW, Pearson TW (2003) The relationship between flesh quality and numbers of *Kudoa**thyrsites* plasmodia and spores in farmed Atlantic salmon, *Salmo**salar*. J Fish Dis 26:451–459. 10.1046/j.1365-2761.2003.00477.x 14513969 10.1046/j.1365-2761.2003.00477.x

[CR8] de Buron I, Hill-Spanik KM, Haselden L, Atkinson SD, Hallett SL, Arnott SA (2017) Infection dynamics of *Kudoa**inornata* (Cnidaria: Myxosporea) in spotted seatrout *Cynoscion**nebulosus* (Teleostei: Sciaenidae). Dis Aquat Org 127:29–40. 10.3354/dao03174 10.3354/dao03174 29256425

[CR9] Dyková I, de Buron I, Fiala I, Roumillat WA (2009). *Kudoa inornata* sp. n. (Myxosporea: Multivalvulida) from the skeletal muscles of *Cynoscion nebulosus* (Teleostei: Sciaenidae). Folia Parasitol 56:91–98. 10.14411/fp.2009.014 10.14411/fp.2009.014 19606785

[CR10] Giulietti L, Karlsbakk E, Cipriani P, Bao M, Storesund JE, Marathe NP, Levsen A (2022) Long-term investigation of the ‘soft flesh’ condition in Northeast Atlantic mackerel induced by the myxosporean parasite *Kudoa**thyrsites* (Cnidaria, Myxozoa): temporal trends and new molecular epidemiological observations. Fish Res 248:106221. 10.1016/j.fishres.2021.106221

[CR11] Hallett SL, Bartholomew JL (2006) Application of a real-time PCR assay to detect and quantify the myxozoan parasite *Ceratomyxa**shasta* in river water samples. Dis Aquat Org 71(2):109–118. 10.3354/dao071109 10.3354/dao071109 16956058

[CR12] Hallett SL, Atkinson SD, Bartholomew JL, Székely C (2015) Myxozoans exploiting homeotherms. In: Okamura B, Gruhl A, Bartholomew J (eds) Myxozoan evolution, ecology and development. Springer, Cham, pp 125–135. 10.1007/978-3-319-14753-6_7

[CR13] Hamilton BR, Marshall DL, Casewell NR, Harrison RA, Blanksby SJ, Undheim EAB (2020) Mapping enzyme activity on tissue by functional mass spectrometry imaging. Angew Chem Int Ed Engl 59:3855–3858. 10.1002/anie.201911390 31854493 10.1002/anie.201911390 PMC7106485

[CR14] Hammami I, Nuel G, Garcia A (2013) Statistical properties of parasite density estimators in malaria. PLoS ONE 8:e51987. 10.1371/journal.pone.0051987 23516389 10.1371/journal.pone.0051987 PMC3597708

[CR15] Henning SS, Hoffman LC, Manley M (2013) A review of *Kudoa*-induced myoliquefaction of marine fish species in South Africa and other countries. S Afr J Sci 109:1–5. 10.1590/sajs.2013/20120003

[CR16] Kabata Z, Whitaker DJ (1981) Two species of *Kudoa* (Myxosporea: Multivalvulida) parasitic in the flesh of *Merluccius**productus* (Ayres, 1855) (Pisces: Teleostei) in the Canadian Pacific. Can J Zool 59:2085–2091. 10.1139/z81-285

[CR17] Kawai T, Sekizuka T, Yahata Y, Kuroda M, Kumeda Y, Iijima Y, Kamata Y, Sugita-Konishi Y, Ohnishi T (2012) Identification of *Kudoa**septempunctata* as the causative agent of novel food poisoning outbreaks in Japan by consumption of *Paralichthys**olivaceus* in raw fish. Clin Infect Dis 54:1046–1052. 10.1093/cid/cir1040 22281845 10.1093/cid/cir1040

[CR18] Kirk D, Jones N, Peacock S, Phillips J, Molnár PK, Krkošek M, Luijckx P (2018) Empirical evidence that metabolic theory describes the temperature dependency of within-host parasite dynamics. PLoS Biol 16:e2004608. 10.1371/journal.pbio.2004608 29415043 10.1371/journal.pbio.2004608 PMC5819823

[CR19] Kudo G, Barnett HJ, Nelson RW (1987) Factors affecting cooked texture quality of Pacific whiting, *Merluccius productus*, fillets with particular emphasis on the effects of infection by the myxosporeans *Kudoa paniformis* and *K. thyrsitis* [*sic*]. Fish Bull 85:745–756. https://spo.nmfs.noaa.gov/sites/default/files/pdf-content/1987/854/kudo.pdf

[CR20] Lüdecke et al (2021) performance: an R package for assessment, comparison and testing of statistical models. J Open Source Softw 6:3139. 10.21105/joss.03139

[CR21] Manley CB, Rakocinski CF, Lee PG, Blaylock RB (2015) Feeding frequency mediates aggression and cannibalism in larval hatchery-reared spotted seatrout, *Cynoscion**nebulosus*. Aquaculture 437:155–160. 10.1016/j.aquaculture.2014.11.012

[CR22] McElroy EJ, George A, de Buron I (2015) The muscle dwelling myxozoan, *Kudoa**inornata*, enhances swimming performance in the spotted seatrout, *Cynoscion**nebulosus*. Parasitol Res 114:2451–2457. 10.1007/s00436-015-4441-z 25876046 10.1007/s00436-015-4441-z

[CR23] O’Connor MI, Bernhardt JR (2018) The metabolic theory of ecology and the cost of parasitism. PLoS Biol 16(4):e2005628. 10.1371/journal.pbio.2005628 29608559 10.1371/journal.pbio.2005628 PMC5897036

[CR24] Oliva M, Luque JL, Teran L, Llican L (1992) *Kudoa**sciaenae* (Myxozoa: Multivalvulidae) cysts distribution in the somatic muscles of *Stellifer**minor* (Tschudi, 1844) (Pisces: Sciaenidae). Mem Inst Oswaldo Cruz 87:33–35. 10.1590/S0074-02761992000100006

[CR25] Pedersen TL (2022) Patchwork: the composer of plots. R package version 1.1.2. https://CRAN.Rproject.org/package=patchwork

[CR26] Pedersen TL, Shemanarev M (2022) Ragg: graphic devices based on AGG. R package version 1.2.3. https://CRAN.R-project.org/package=ragg

[CR27] Pinheiro J, Bates D, DebRoy S, Sarkar D, R Core Team (2021) Nlme: linear and nonlinear mixed effects models. R package version 3.1–153. https://CRAN.R-project.org/package=nlme

[CR28] Revelle W (2024) Psych: procedures for psychological, psychometric, and personality research. Northwestern University, Evanston, Illinois. R package version 2.4.3. https://CRAN.R-project.org/package=psych

[CR29] R Core Team (2022) R: a language and environment for statistical computing. R foundation for statistical computing. Vienna, Austria. https://www.R-project.org/

[CR30] Snyder AM, McElroy EJ, Smith JF, Archambault J, de Buron I (2022) Limited accrual of myxospores of *Kudoa**inornata* (Cnidaria: Myxosporea) in their wild fish hosts, *Cynoscion**nebulosus* (Teleostei: Sciaenidae). Dis Aquat Org 151:51–60. 10.3354/dao03689 10.3354/dao03689 36106716

[CR31] Sokal RR, Rohlf FJ (1995) Biometry. W.H.Freeman and Company, New York

[CR32] Stadler E, Cromer D, Ogunlade S, Ongoiba A, Doumbo S, Kayentao K, Traore B, Crompton P D, Portugal S, Davenport MP, Khoury DS (2023) Evidence for exposure dependent carriage of malaria parasites across the dry season: modelling analysis of longitudinal data.Malar J 22. 10.1186/s12936-023-04461-1 10.1186/s12936-023-04461-1 PMC9898990 36737743

[CR33] Ware S, Roumillat WA, Connors VA, de Buron I (2014) Distribution of *Kudoa**inornata* plasmodia in the musculature of its host, the spotted seatrout *Cynoscion**nebulosus*. Comp Parasitol 81:10–14. 10.1654/4668.1

[CR34] Wickham H (2016) ggplot2: elegant graphics for data analysis. Springer-Verlag, New York

[CR35] Wickham H, Seidel D (2020) Scales: scale functions for visualization. R package version 1.1.1. https://CRAN.R-project.org/package=scales

[CR36] Wickham H, François R, Henry L, Müller K, Vaughan D (2023) dplyr: a grammar of data manipulation. R package version 1.1.2. https://CRAN.R-project.org/package=dplyr

[CR37] Yokoyama H, Lu M, Mori KI, Satoh J, Mekata T, Yoshinaga T (2015) Infection dynamics of *Kudoa**septempunctata* (Myxozoa: Multivalvulida) in hatchery-produced olive flounder *Paralichthys**olivaceus*. Fish Pathol 50:60–67. 10.3147/JSFP.50.60

